# Normal Saline in Septic Cases

**Published:** 1900-04-21

**Authors:** 


### NORMAL SALINE IN SEPTIC CASES.

In conjunction with the above article it is not without interest to refer to a paper recently read before a meeting of a medical association at Charlestown, U.S.A., by Dr. Virginius Harrison,[1] Lecturer on the Practice of Surgery in the University College, Richmond, in which, among other uses of the normal saline solution, he instances its employment in the treatment of sepsis. He does not seem to mind whether it is given in the form of repeated enemata, or as a hypodermic, or an intra-venous infusion. In most of his cases, however, it was administered as an enema. Among the instances which he relates was one of profound sepsis following an operation for double pyo-salpinx. For a week the patient's temperature ranged from 104 deg. Fahr. to 106 deg. Fahr. After resorting to various other measures Dr. Harrison tried the rectal injection of a pint of a saline solution, and repeated the dose every four hours. "As a result there was a fall in the temperature to 100 deg. Fahr. within 24 hours. The enemata were continued until the acute symptoms had abated, and the patient was well on the road to recovery."

Another case was one of localised suppurative peritonitis. The abdomen had been opened and drained, yet the pulse remained rapid and the temperature high. "The salines were used as in the former case with perfect elimination of the poison and the recovery of the patient." He goes on to say that in puerperal sepsis "the results obtained are as certain, prompt, and as effective as in surgical sepsis." Unless the source of the infection be removed in either case the benefit derived may be only temporary, but "we can certainly in both diminish the intensity of the infection until more radical measures can be utilised." How these things act it is not easy to say—indeed, even theorising partakes more or less of guesswork—but in view of the beneficial effects which, for a time at least, follow saline infusion in cases of uræmia, and in some cases of diabetic coma, both of them conditions in which the blood is charged to a poisonous degree with toxic matters, it seems only reasonable to assume that the tissues are better able to deal with such poison in a dilute than in a concentrated form; and as what is called puerperal septicæmia is often a toxæmia rather than an infection this may possibly be the explanation of the good effects of saline infusion in these cases. Whether in some of the reported cases in which the injection of sera has seemed to do so much good the material used has acted in the same way, rather than by its special antitoxic virtue, is another question into which it would be premature to enter at the present moment; but it is clear enough that in view of the multiplicity of microbes, whose products go to produce the condition known as sepsis, there is but little certainty that the right serum has always been used, notwithstanding the good results which have sometimes followed.

[1] Charlotte Med. Jour., Mar., 1900.

